# Supplementary material for: Repurposing clinically safe drugs for DNA repair pathway choice in CRISPR genome editing and synthetic lethality
Source: Nat Commun. 2025 Dec 10;16:11077. doi: 10.1038/s41467-025-67243-0 (PMC12698765; doi:10.1038/s41467-025-67243-0)
Supplement: Supplementary file 1 — Supplementary Information [file 41467_2025_67243_MOESM1_ESM.pdf]

Supplementary Information

Repurposing clinically safe drugs for DNA repair pathway choice in CRISPR genome editing and synthetic lethality

Dominik Macak[#1], Philipp Kanis[#1], Stephan Riesenberg*[1]

Affiliations

[1]Max Planck Institute for Evolutionary Anthropology, Leipzig, Germany

[#]Authors contributed equally.

*Corresponding author: stephan_riesenberg@eva.mpg.de

Supplementary Figures

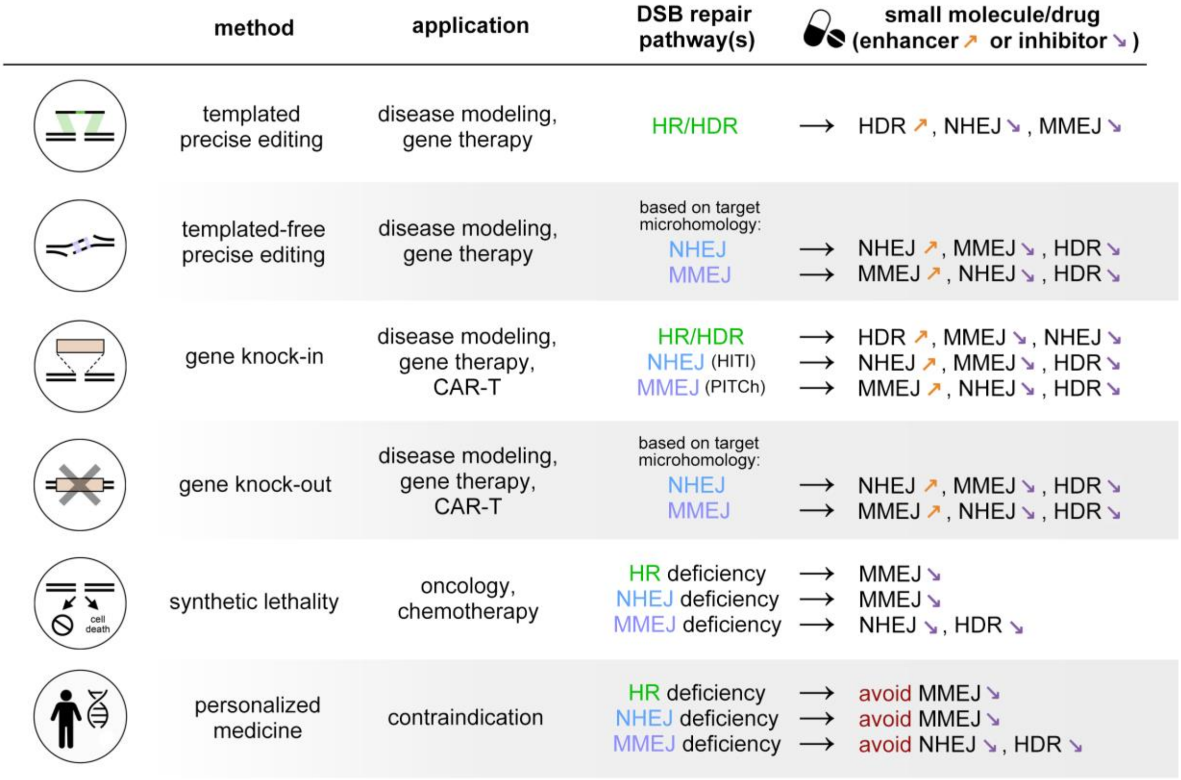

Supplementary Fig. 1 | Potential clinically relevant applications of small molecules/repurposed drugs that modulate DSB repair. For each method, related application(s), utilized DSB repair pathways and impact of small molecule enhancers and inhibitors of DSB repair pathways are shown. CAR-T: chimeric antigen receptor T-cell; HITI: homology-independent targeted integration; PITCh: precise integration into target chromosome.

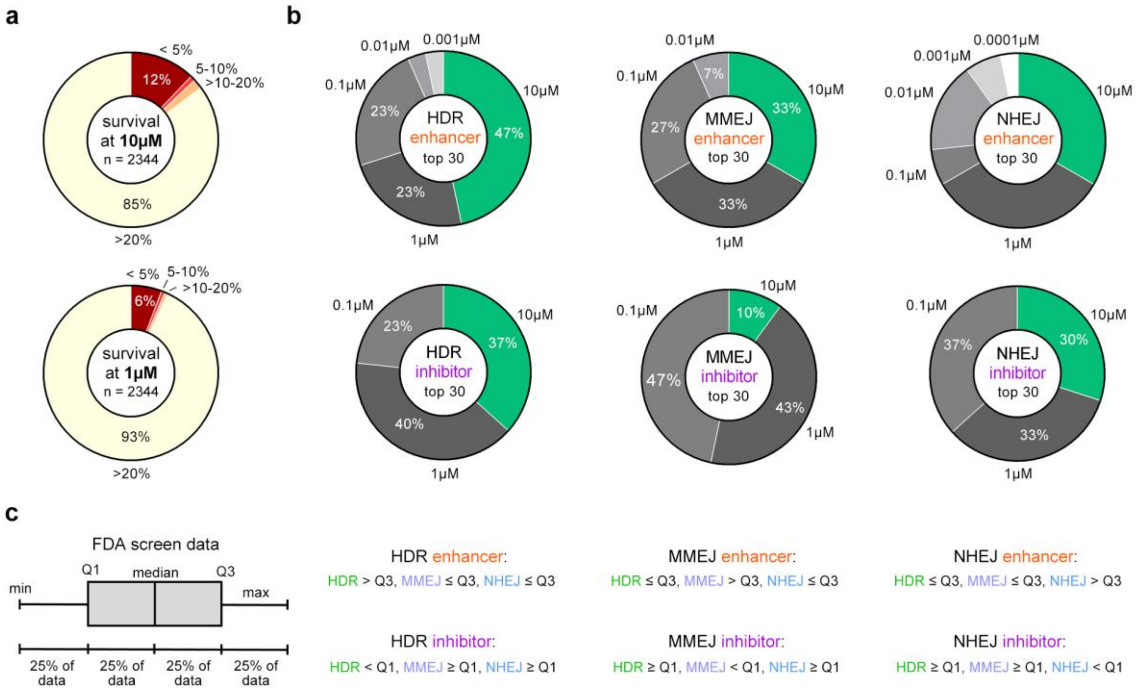

Supplementary Fig. 2 | Library wide concentration-dependent drug effects and DSB repair modulator categorization. (**a**) Share of drugs tested in the initial drug screen at a concentration of 10µM (upper pie chart) and of 1µM (lower pie chart) that exhibit survival lower than 5% (dark red), 5-20% (red, orange), and higher than 20% (light yellow). (**b**) Share of tested drug concentrations among the top 30 compounds categorized as enhancers or inhibitors of outcomes attributed to HDR, NHEJ, and MMEJ, respectively. Drug hits at 10µM (green) could be missed when screening drugs only at 1µM. (**c**) Categorization of DSB repair pathway outcome enhancers or inhibitors based on quartiles Q1 and Q3 of the FDA screen data (see also Methods). Source data are provided as a Source Data file.

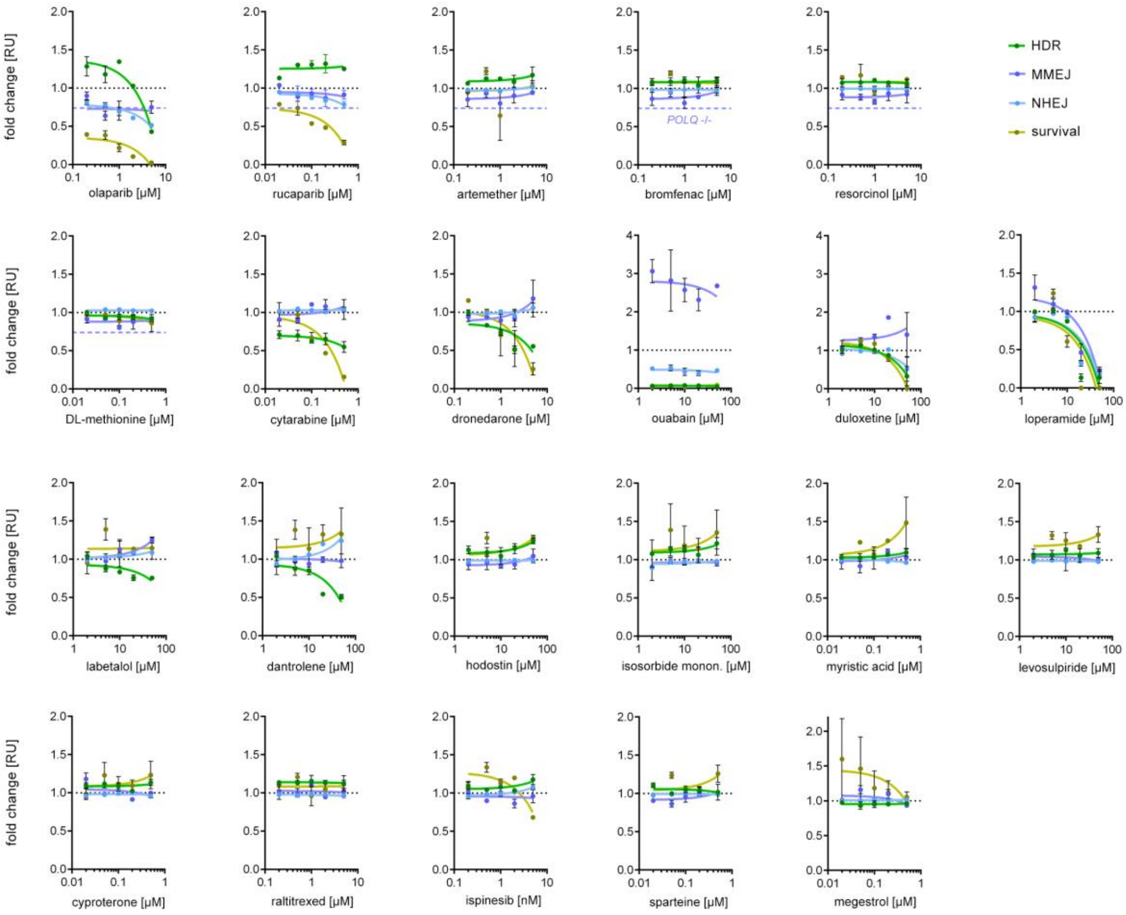

Supplementary Fig. 3 | Genome editing and survival dose-response curves of repurposed drugs. Dose-response curves related to Fig. 2c. For each metric the linear regression across concentrations is shown. Dots represent the mean of independent biological replicates (n = 2), and error bars show the s.e.m. For drugs reducing MMEJ the full inhibition of MMEJ by *POLQ* knock-out[1] (0.74) is shown as purple dashed line. Source data are provided as a Source Data file.

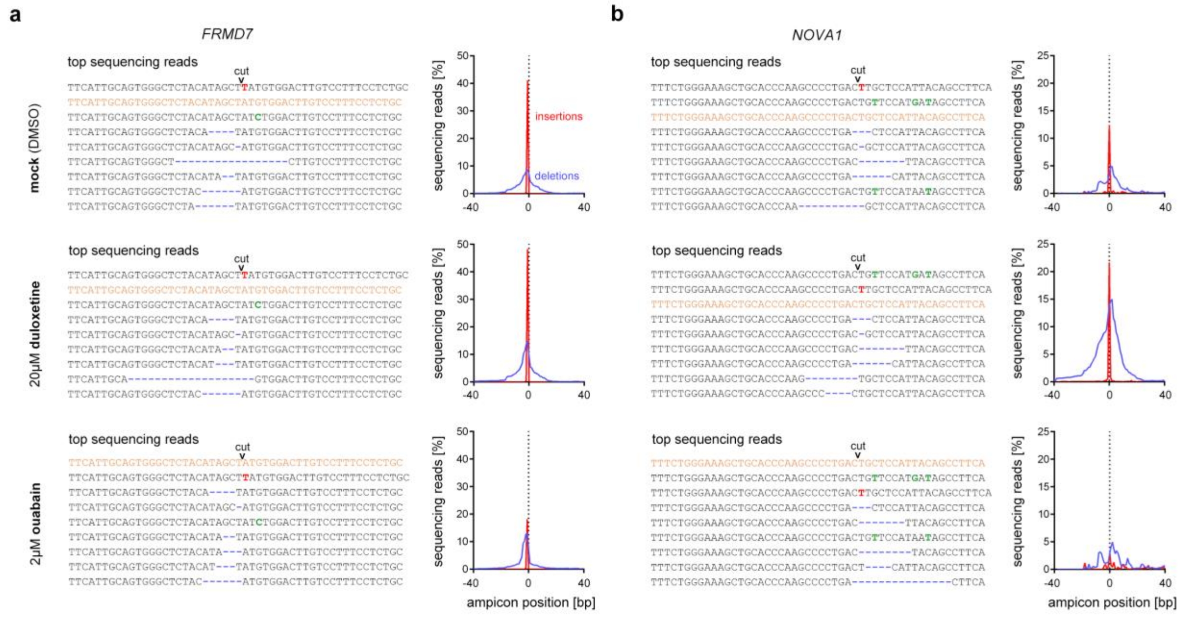

Supplementary Fig. 4 | Influence of MMEJ enhancers on indel patterns. Top sequencing reads (left panels) and frequencies of insertions and deletions (right panels) at the *FRMD7* target (**a**) and *NOVA1* target (**b**) in 409B2 hiPSCs, when editing without a drug (top panels), 20µM duloxetine (middle panels), or 2µM ouabain (bottom panels). The wild type sequence is given in orange. Red, blue, or green bases indicate an insertion, deletion, or precise edits, respectively. The blue deletion line and red insertion line is the mean of independent biological replicates (n = 6). The vertical dotted line indicates the position of the cut site. Data shown is related to Fig. 2d. Source data are provided as a Source Data file.

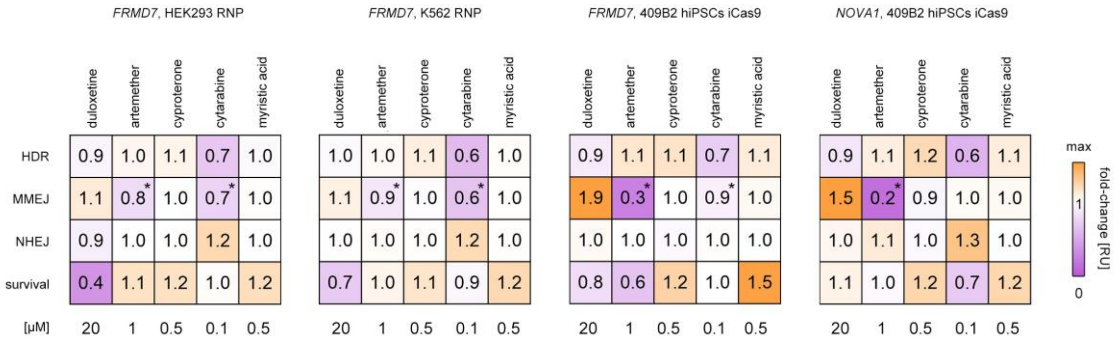

Supplementary Fig. 5 | Repurposed DSB repair modulator drugs in different cell types. Heatmap of selected drugs with relative fold-changes of HDR, MMEJ, NHEJ, after editing in HEK293 and K562 cells. For ease of comparison, data from Fig. 2 in iPSCs is also shown. Asterisks indicate normalized MMEJ changes based on the method's detection limit for MMEJ reduction (full inhibition = 0.74 for *POLQ* knock-out[1]). Independent biological replicates were performed (n = 6, except n = 2 for *FRMD7* in hiPSCs).

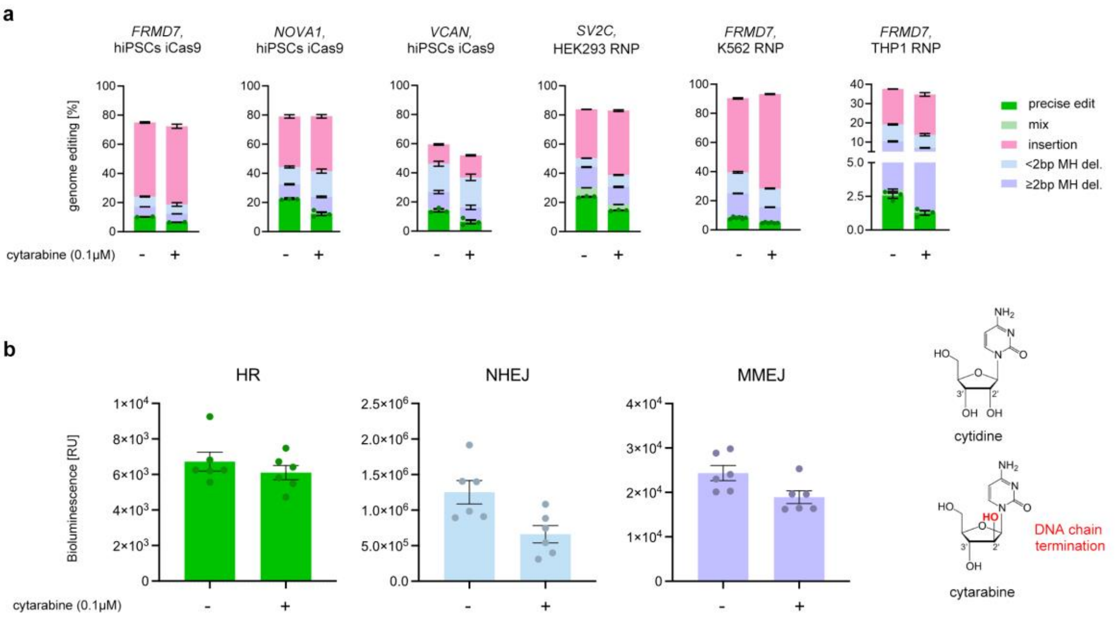

Supplementary Fig. 6 | Impact of cytarabine on genome editing outcomes. (**a**) Genome editing efficiencies with or without cytarabine for *FRMD7, NOVA1*, and *VCAN* in iCas9 hiPSCs, and using Cas9-HiFi RNP for *SV2C* in HEK293 cells, as well as *FRMD7* in K562 and THP1 cells. Independent biological replicates were performed (n = 3, except n = 2 for *FRMD7* in hiPSCs and n = 6 for *FRMD7* in K562) and error bars indicate the s.e.m. For precise edits, replicates are depicted by dots. (**b**) Extrachromosomal DSB repair reporter assays to assess the effect of cytarabine on HR, NHEJ, and MMEJ. 24 hours after transfection and treatment, luciferase activity was quantified. NanoLuc luciferase bioluminescence values were normalized to the firefly luciferase bioluminescence of the wild type condition with (non-targeting siRNA) or without siRNA treatment. Independent replicates were performed (n = 6) and error bars show the s.e.m. Source data are provided as a Source Data file.

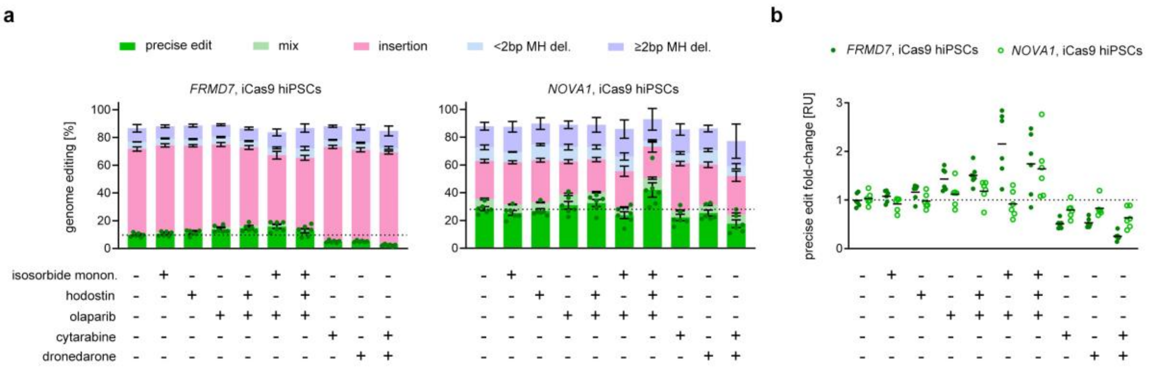

Supplementary Fig. 7 | Effect of HDR outcome modulator drug combinations on genome editing. (**a**) Genome editing efficiencies for combinations of drugs in 409B2 iCRISPR Cas9 hiPSCs targeting *FRMD7* and *NOVA1*. Independent biological replicates were performed (n = 6). Error bars indicate the s.e.m. For precise edits replicates are depicted as dots. (**b**) Relative fold-change of precise edits (from a) for combinations drugs in 409B2 iCRISPR Cas9 hiPSCs targeting *FRMD7 (*filled green circles) and *NOVA1* (open green circles). Source data are provided as a Source Data file.

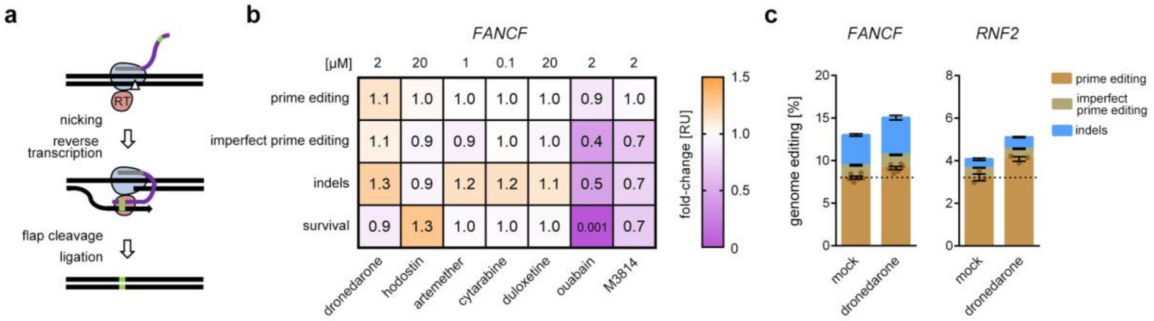

Supplementary Fig. 8 | Influence of repurposed drugs on prime editing. (**a**) Schematic of prime editing. Prime editing relies on the introduction of single-strand breaks by a nickase, which is linked to a reverse transcriptase (RT), that uses the cleaved strand of the target site as a primer to introduce edits from a prime editing gRNA (pegRNA). Flap cleavage is followed by ligation and mismatch repair to copy the edit in the second DNA strand. (**b**) Heatmap of relative fold-changes of prime editing, imperfect prime editing, indels, and survival using an inducible Cas9H840A nickase prime editor in 409B2 hiPSCs to edit *FANCF* (+5G to T) using different drugs. (**c**) Absolute genome editing efficiencies for prime editing of *FANCF* (+5G to T) and *RNF2* (+1C to A) with and without 2µM dronedarone. Independent biological replicates were performed (n = 6 for *FANCF*, n = 3 for *RNF2*) and error bars show the s.e.m. Replicates are depicted by dots. Source data are provided as a Source Data file.

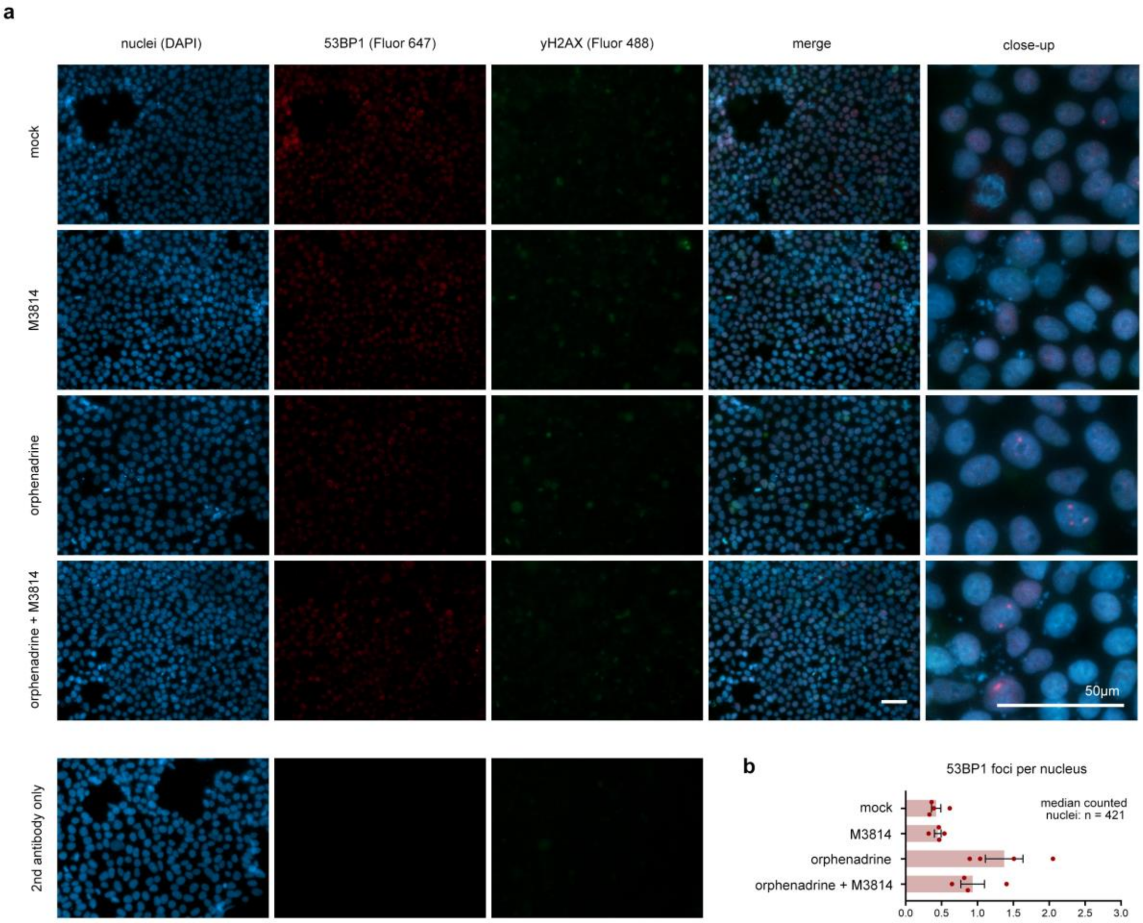

Supplementary Fig. 9 | Immunostainings after orphenadrine treatment. (**a**) Representative immunostaining images of 53BP1 (Fluor 647), γ-H2AX (Fluor 488), and nuclei counterstain of wild type hiPSCs with and without treatment by 10µM orphenadrine and/or 2µM M3814 for 24h. Close-up images were equally increased for brightness and contrast. (**b**) Quantification of 53BP1 foci per nucleus corresponding to a. Dots indicate counts from different from independent replicates and the median number of counted nuclei across all conditions is stated. Independent biological replicates were performed (n = 4). Error bars indicate the s.e.m. Source data are provided as a Source Data file.

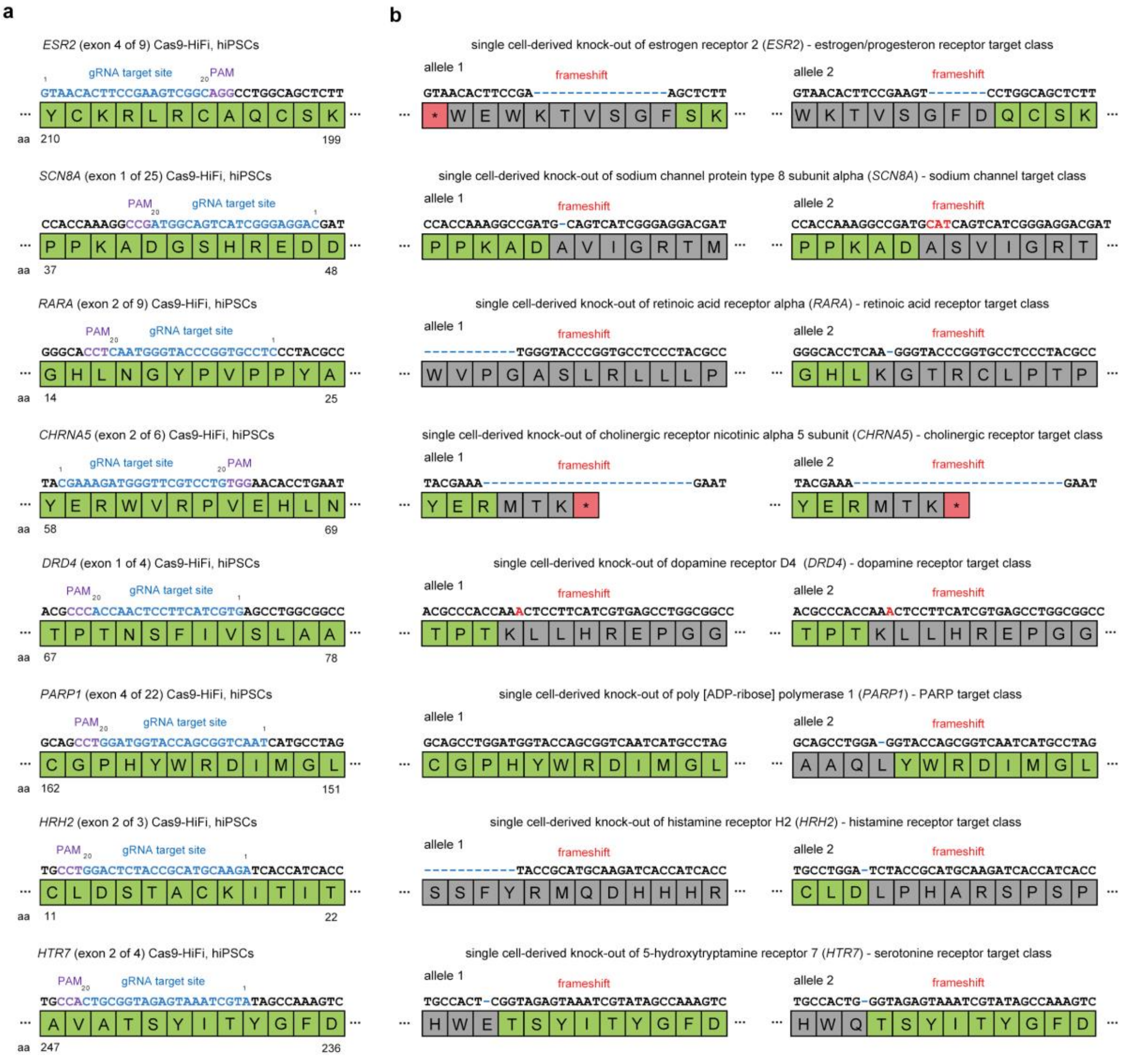

Supplementary Fig. 10 | Genotypes of single cell-derived knock-out clones. (**a**) Indicated are the genomic loci of the representative gene with the DNA sequence and the corresponding amino acid translation. gRNA and protospacer adjacent motif (PAM) sequence are highlighted in blue and purple, respectively. (**b**) Outcome of CRISPR-based knock-out per allele in single cell-derived clones. Nucleotide deletions are indicated by a dash. DNA mutations are highlighted in red and resulting frame shift or knockouts on the amino acid sequence are indicated.

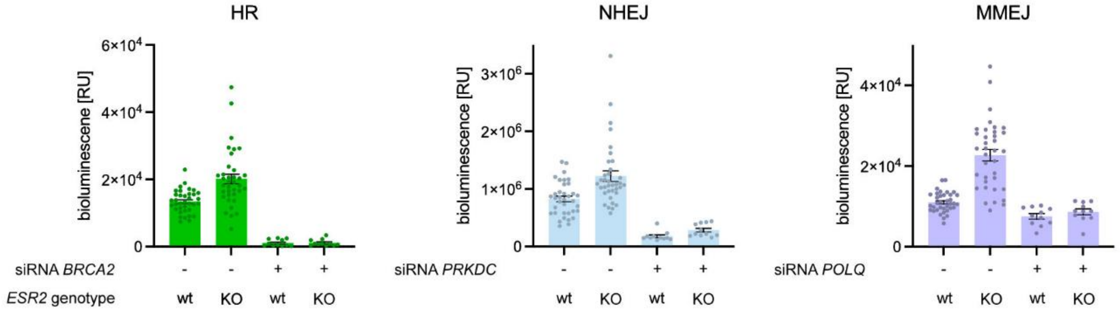

Supplementary Fig. 11 | Extrachromosomal DSB repair reporter assays to assess the effect of *ESR2* knock out on HR, NHEJ, and MMEJ. 409B2 hiPSCs with or without *ESR2* knockout were electroporated with siRNA (*BRCA2*, *PRKDC*, *POLQ*, or non-targeting) two days prior to transfection of the reporter assay components. 24h after transfection, luciferase activity was quantified. NanoLuc luciferase bioluminescence values were normalized to the firefly luciferase bioluminescence of the wild type condition with (non-targeting siRNA) or without siRNA treatment. Independent replicates were performed (n = 36 for non-targeting siRNA, n = 13 for targeting siRNA) and error bars show the s.e.m. Source data are provided as a Source Data file.

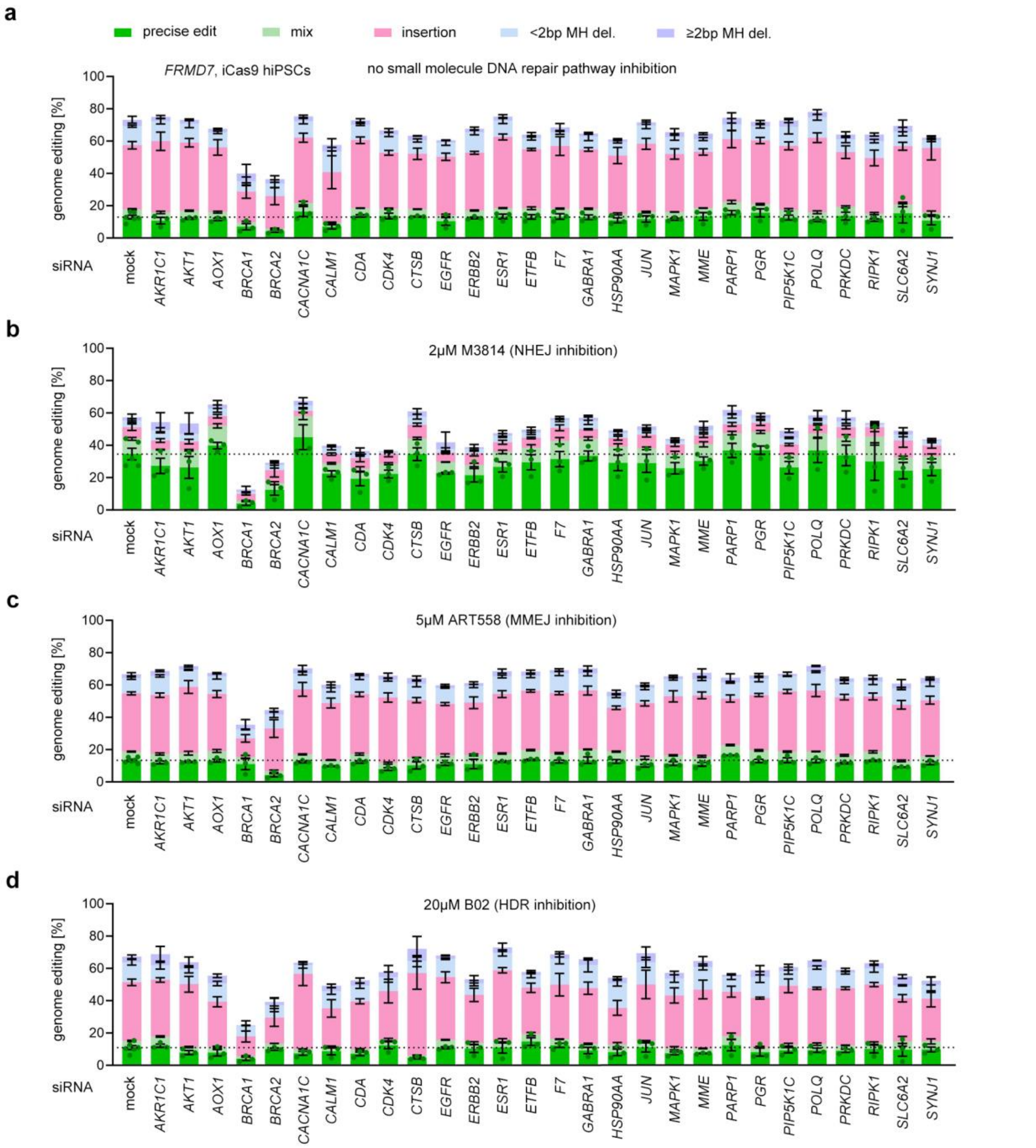

Supplementary Fig. 12 | Effect of STRING candidate siRNAs and combined small molecule repair pathway inhibition on genome editing. Genome editing efficiencies for *FRMD7* in 409B2 iCas9 hiPSCs by lipofection of oligonucleotides with no small molecule repair pathway inhibition (**a**), NHEJ inhibition by 2µM M3814 (**b**), MMEJ inhibition by 5µM ART558 (**c**), and HDR inhibition by 20µM B02 (**d**). siRNAs were selected to target mRNAs of 25 STRING-predicted genes, or control genes central to HR (*BRCA2*), NHEJ (*PRKDC*), and MMEJ (*POLQ*). Independent biological replicates were performed (n = 6 for mock and n = 3 for siRNAs for a-d). Error bars indicate the s.e.m. Source data are provided as a Source Data file.

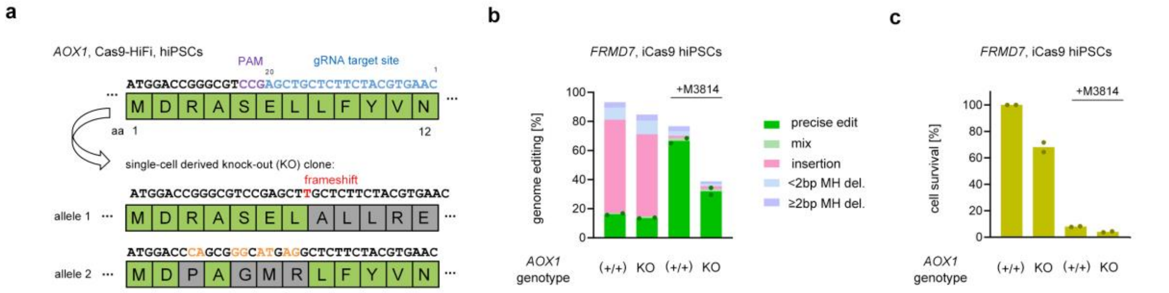

Supplementary Fig. 13 | Generation and characterization of *AOX1* knock-out in hiPSCs. (**a**) Generation of an *AOX1* knock-out hiPSC line. Cas9-HiFi was targeted to a protospacer adjacent motif (PAM) within the *AOX1* coding sequence using indicated gRNA. A single-cell-derived clone exhibited a frameshift mutation in allele 1 and four amino acid substitutions in allele 2, resulting in an at least monoallelic knock-out. (**b**) Genome editing efficiencies of *FRMD7* in wild type and *AOX1* knock-out iCas9 hiPSCs, with or without M3814. Independent biological replicates were performed (n = 2). For precise edits, replicates are depicted by dots. (**c**) Cell survival corresponding to the genome editing experiments in b. Source data are provided as a Source Data file.

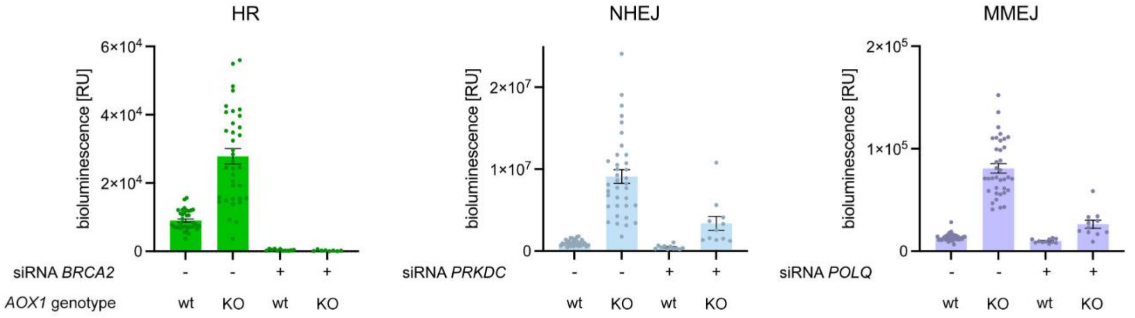

Supplementary Fig. 14 | Extrachromosomal DSB repair reporter assay to assess the effect of *AOX1* knock out on HR, NHEJ, and MMEJ. 409B2 hiPSCs with or without an *AOX1* knockout were electroporated with siRNA (*BRCA2*, *PRKDC*, *POLQ*, or non-targeting) two days prior to transfection of the reporter assay components. 24h after transfection, luciferase activity was quantified. NanoLuc luciferase bioluminescence values were normalized to the firefly luciferase bioluminescence of the wild type condition with (non-targeting siRNA) or without siRNA treatment. Independent replicates were performed (n = 36 for non-targeting siRNA, n = 13 for targeting siRNA) and error bars show the s.e.m. Source data are provided as a Source Data file.

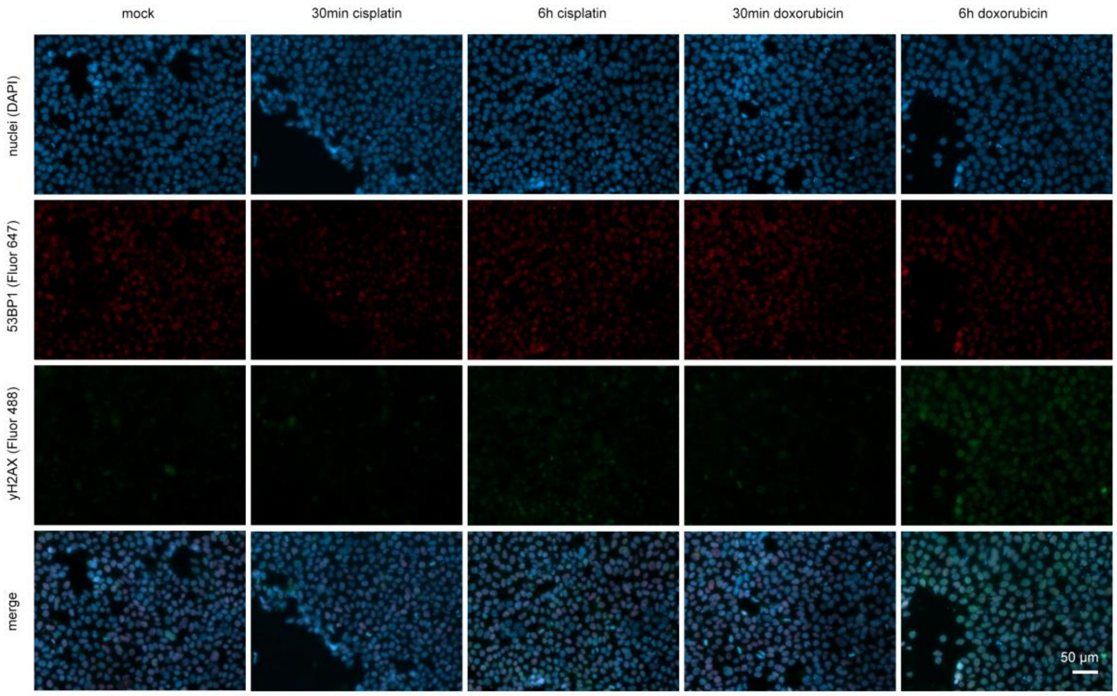

Supplementary Fig. 15 | Immunostaining of wild type hiPSCs. Representative immunostaining images of 53BP1 (Fluor 647), γ-H2AX (Fluor 488), and nuclei counterstain after treatment with and without 2µM cisplatin, or 1µM doxorubicin for 30min and 6h. Quantification of 53BP1 foci per nucleus and contrast enhanced close-up images are shown in Fig. 4g. Eight images for untreated cells and four images for treated cells from independent biological replicates were used for quantification.

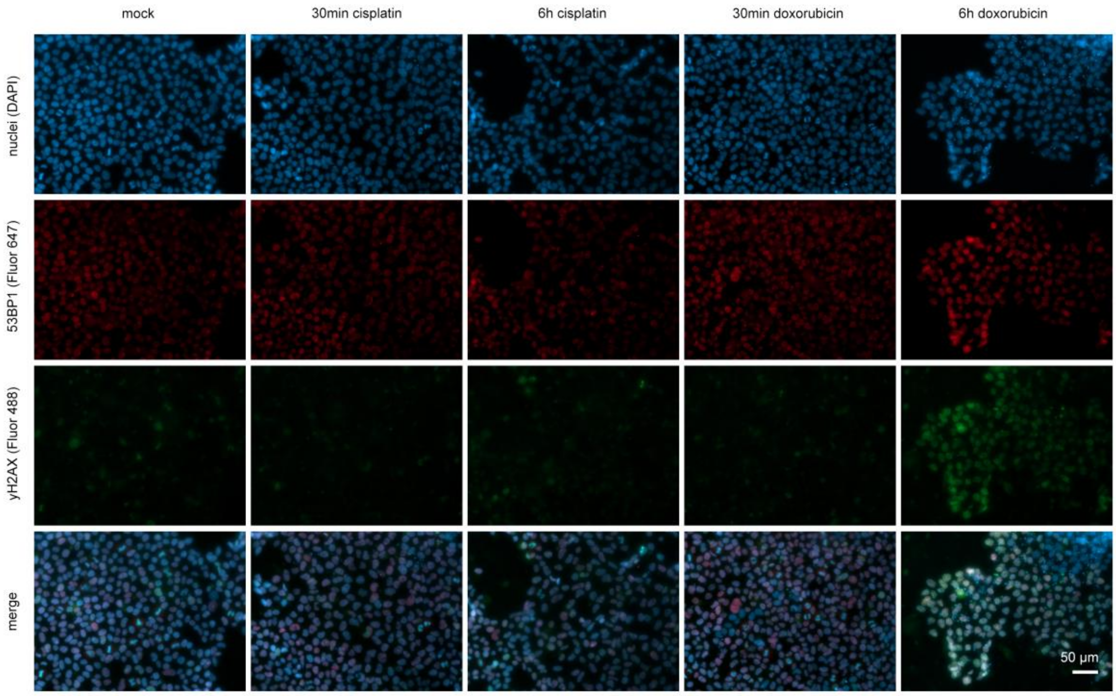

Supplementary Fig. 16 | Immunostaining of *ESR2* knock-out hiPSCs. Representative immunostaining images of 53BP1 (Fluor 647), γ-H2AX (Fluor 488), and nuclei counterstain after treatment with and without 2µM cisplatin, or 1µM doxorubicin for 30min and 6h. Quantification of 53BP1 foci per nucleus and contrast enhanced close-up images are shown in Fig. 4g. Eight images for untreated cells and four images for treated cells from independent biological replicates were used for quantification.

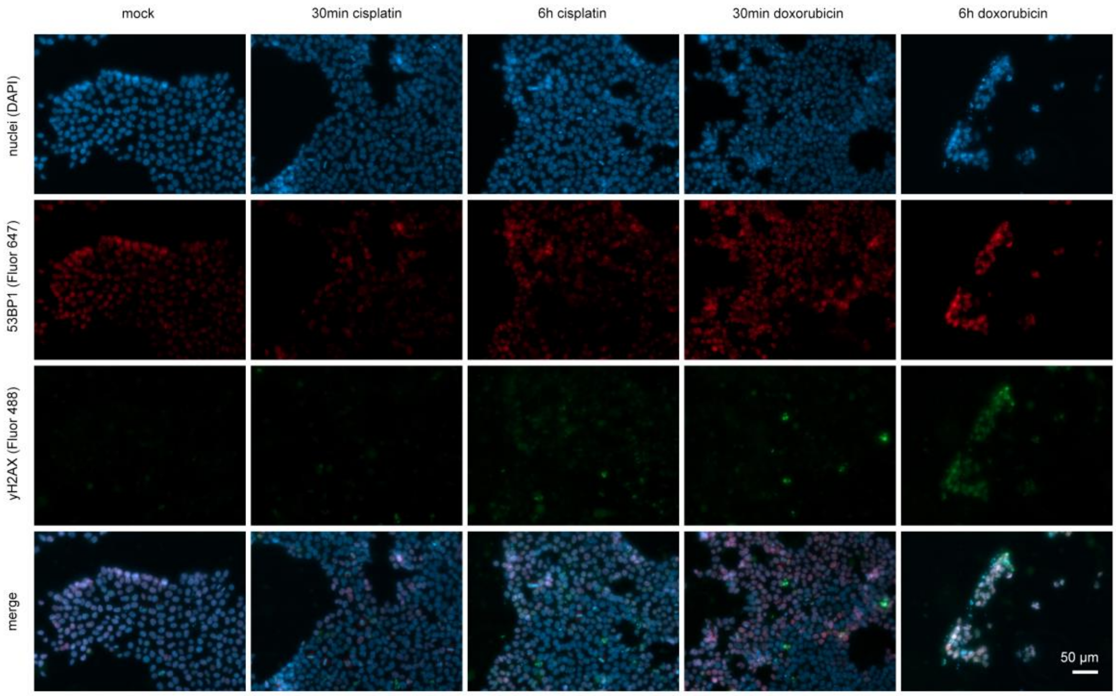

Supplementary Fig. 17 | Immunostaining of *AOX1* knock-out hiPSCs. Representative immunostaining images of 53BP1 (Fluor 647), γ-H2AX (Fluor 488), and nuclei counterstain after treatment with and without 2µM cisplatin, or 1µM doxorubicin for 30min and 6h. Quantification of 53BP1 foci per nucleus and contrast enhanced close-up images are shown in Fig. 4g. Eight images for untreated cells and four images for treated cells from independent biological replicates were used for quantification.

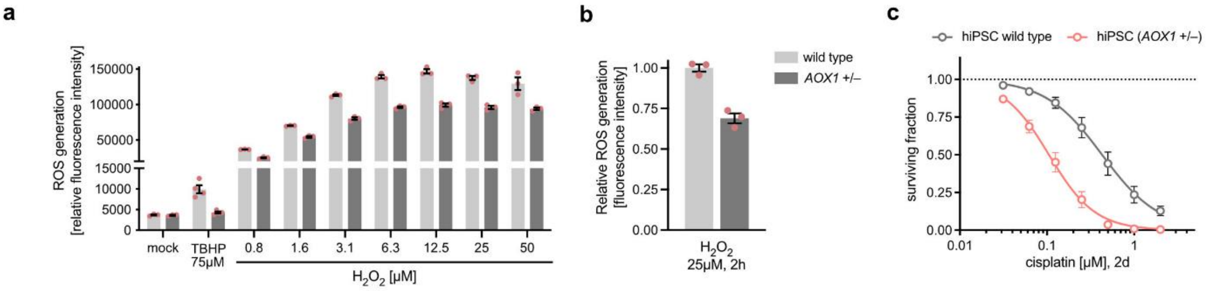

Supplementary Fig. 18 | Effect of AOX1 on cellular and DNA damage stress. (**a**) Reactive oxygen species production in wild type and AOX1-deficient cells. 409B2 hiPSCs were stained with 2',7'-dichlorfluorescein diacetate (DCFDA) and subsequently treated with 75µM tert-butyl hydroperoxide (TBHP) or H_2O_2 (0–50µM) for 2h. ROS production was determined by the formation of the fluorescent compound 2',7'-dichlorofluorescein. Values were normalized to cell viability of untreated cells. Independent replicates were performed (n = 3, except n = 4 for TBHP). Bars and errors indicate the mean and s.e.m. (**b**) Reactive oxygen species in wild type and AOX1-deficient 409B2 cells after 2h treatment with 25µM H_2O_2. Bars and errors indicate the mean and s.e.m of independent biological replicates (n = 3). (**c**) Dose-response curves of wild type and AOX1-deficient 409B2 hiPSCs to cisplatin treatment after 2d. Dots and errors indicate the mean and s.e.m of independent biological replicates (n = 3). Sigmoidal fits are shown as lines.

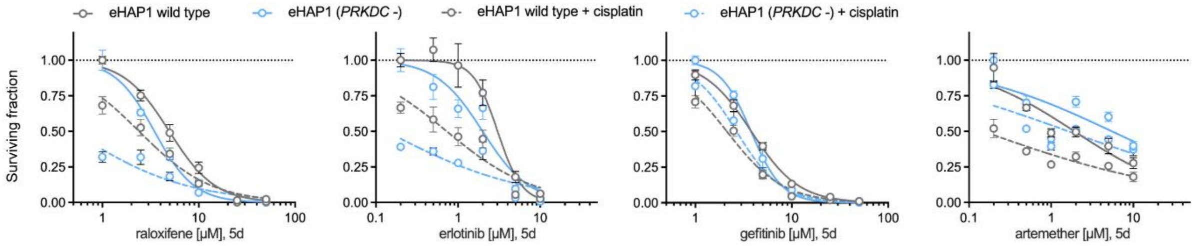

Supplementary Fig. 19 | Synthetic lethality in eHAP1 cells by repurposed drugs. Dose-response curves of surviving fractions in wild type (grey) and *PRKDC*-inactivated eHAP1 cells (blue), with (dashed lines) or without (solid lines) cisplatin (121nM) treatment for 5d. Sigmoidal fits are shown as lines. Independent biological replicates were performed (n = 3) and error bars show the s.e.m. Source data are provided as a Source Data file.

Supplementary References

1 Riesenberg, S. *et al.* Efficient high-precision homology-directed repair-dependent genome editing by HDRobust. *Nature Methods* **20**, 1388-1399 (2023). https://doi.org/10.1038/s41592-023-01949-1
