# Supplementary material for: Repurposing clinically safe drugs for DNA repair pathway choice in CRISPR genome editing and synthetic lethality
Source: Nat Commun. 2025 Dec 10;16:11077. doi: 10.1038/s41467-025-67243-0 (PMC12698765; doi:10.1038/s41467-025-67243-0)
Supplement: Supplementary file 2 — Description of Additional Supplementary Files [file 41467_2025_67243_MOESM2_ESM.pdf]

Description of Additional Supplementary Files

File Name: Supplementary Data 1
Description: FDA drug DSB repair catalog

File Name: Supplementary Data 2
Description: Improved FDA screen layout

File Name: Supplementary Data 3
Description: Subset Screen

File Name: Supplementary Data 4
Description: Functional enrichment

File Name: Supplementary Data 5
Description: List of oligonucleotides
