# Supplementary material for: Repurposing clinically safe drugs for DNA repair pathway choice in CRISPR genome editing and synthetic lethality
Source: Nat Commun. 2025 Dec 10;16:11077. doi: 10.1038/s41467-025-67243-0 (PMC12698765; doi:10.1038/s41467-025-67243-0)
Supplement: Supplementary file 8 — Reporting Summary [file 41467_2025_67243_MOESM8_ESM.pdf]

nature portfolio

Corresponding author(s): Stephan Riesenberg

Last updated by author(s): Nov 14, 2025

Reporting Summary

Nature Portfolio wishes to improve the reproducibility of the work that we publish. This form provides structure for consistency and transparency in reporting. For further information on Nature Portfolio policies, see our Editorial Policies and the Editorial Policy Checklist.

Statistics

For all statistical analyses, confirm that the following items are present in the figure legend, table legend, main text, or Methods section.

n/a	Confirmed	
☐	☒	The exact sample size (*n*) for each experimental group/condition, given as a discrete number and unit of measurement
☐	☒	A statement on whether measurements were taken from distinct samples or whether the same sample was measured repeatedly
☒	☐	The statistical test(s) used AND whether they are one- or two-sided<br>*Only common tests should be described solely by name; describe more complex techniques in the Methods section.*
☒	☐	A description of all covariates tested
☒	☐	A description of any assumptions or corrections, such as tests of normality and adjustment for multiple comparisons
☐	☒	A full description of the statistical parameters including central tendency (e.g. means) or other basic estimates (e.g. regression coefficient) AND variation (e.g. standard deviation) or associated estimates of uncertainty (e.g. confidence intervals)
☐	☒	For null hypothesis testing, the test statistic (e.g. *F*, *t*, *r*) with confidence intervals, effect sizes, degrees of freedom and *P* value noted<br>*Give P values as exact values whenever suitable.*
☒	☐	For Bayesian analysis, information on the choice of priors and Markov chain Monte Carlo settings
☒	☐	For hierarchical and complex designs, identification of the appropriate level for tests and full reporting of outcomes
☐	☒	Estimates of effect sizes (e.g. Cohen's *d*, Pearson's *r*), indicating how they were calculated

Our web collection on statistics for biologists contains articles on many of the points above.

Software and code

Policy information about availability of computer code

Data collection	No software was used.
Data analysis	CRISPResso (v1), SAMtools (v1.12), GraphPad Prism (v10), Adobe Photoshop (CS5), iBright Analysis Software (Thermo Scientific, version 5.4.0), ImageJ (2.16.0/1.54p)

For manuscripts utilizing custom algorithms or software that are central to the research but not yet described in published literature, software must be made available to editors and reviewers. We strongly encourage code deposition in a community repository (e.g. GitHub). See the Nature Portfolio guidelines for submitting code & software for further information.

Data

Policy information about availability of data

All manuscripts must include a data availability statement. This statement should provide the following information, where applicable:

- Accession codes, unique identifiers, or web links for publicly available datasets
- A description of any restrictions on data availability
- For clinical datasets or third party data, please ensure that the statement adheres to our policy

The sequencing data generated in this study are deposited in the NCBI's Sequence Read Archive (SRA) with the accession code PRJNA1354267 [www.ncbi.nlm.nih.gov/sra/PRJNA1354267]. Data are also deposited in the Dryad database under accession code dryad.1ns1rn970 [doi.org/10.5061/dryad.1ns1rn970]. Source data are provided with this paper.

Research involving human participants, their data, or biological material

Policy information about studies with human participants or human data. See also policy information about sex, gender (identity/presentation), and sexual orientation and race, ethnicity and racism.

Reporting on sex and gender	not applicable
Reporting on race, ethnicity, or other socially relevant groupings	*Please specify the socially constructed or socially relevant categorization variable(s) used in your manuscript and explain why they were used. Please note that such variables should not be used as proxies for other socially constructed/relevant variables (for example, race or ethnicity should not be used as a proxy for socioeconomic status).* *Provide clear definitions of the relevant terms used, how they were provided (by the participants/respondents, the researchers, or third parties), and the method(s) used to classify people into the different categories (e.g. self-report, census or administrative data, social media data, etc.)* *Please provide details about how you controlled for confounding variables in your analyses.*
Population characteristics	*Describe the covariate-relevant population characteristics of the human research participants (e.g. age, genotypic information, past and current diagnosis and treatment categories). If you filled out the behavioural & social sciences study design questions and have nothing to add here, write "See above."*
Recruitment	*Describe how participants were recruited. Outline any potential self-selection bias or other biases that may be present and how these are likely to impact results.*
Ethics oversight	*Identify the organization(s) that approved the study protocol.*

Note that full information on the approval of the study protocol must also be provided in the manuscript.

Field-specific reporting

Please select the one below that is the best fit for your research. If you are not sure, read the appropriate sections before making your selection.

☒ Life sciences ☐ Behavioural & social sciences ☐ Ecological, evolutionary & environmental sciences

For a reference copy of the document with all sections, see nature.com/documents/nr-reporting-summary-flat.pdf

Life sciences study design

All studies must disclose on these points even when the disclosure is negative.

Sample size	No sample size calculation was performed. Except for the high-throughput screen (one replicate per condition), at least two independent biological replicates for each condition were obtained. This was sufficient as the editing efficiency difference between replicates of a respective group was very small compared to the strong difference observed between the compared groups.
Data exclusions	No data were exluded from analysis.
Replication	All attempts of replication were successful. At least two independent biological replicates for each condition were obtained.
Randomization	Mammalian cells used in this study were cells grown under identical conditions and experiments were done in parallel. Thus, no randomization was used.
Blinding	Mammalian cells used in this study were cells grown under identical conditions and experiments were done in parallel. Thus, blinding was not used for data collection. Analysis was performed based on numerical sample names, without the identity of the samples known during the analysis.

Reporting for specific materials, systems and methods

We require information from authors about some types of materials, experimental systems and methods used in many studies. Here, indicate whether each material, system or method listed is relevant to your study. If you are not sure if a list item applies to your research, read the appropriate section before selecting a response.

Materials & experimental systems

n/a	Involved in the study
☐	☒ Antibodies
☐	☒ Eukaryotic cell lines
☒	☐ Palaeontology and archaeology
☒	☐ Animals and other organisms
☒	☐ Clinical data
☒	☐ Dual use research of concern
☒	☐ Plants

Methods

n/a	Involved in the study
☒	☐ ChIP-seq
☒	☐ Flow cytometry
☒	☐ MRI-based neuroimaging

Antibodies

Antibodies used

Primary antibodies for Western Blots were from Cell Signaling: ATM (catalog no. 2873), DNA-PKcs (catalog no. 4602), POLQ (catalog no. 64708), p-BRCA1 (catalog no. 9009), BRCA2 (catalog no. 10741), LIG4 (catalog no. 14649), HSP27 (catalog no. 2402). The secondary antibody for Western blots was a horseradish peroxidase (HRP)-conjugated goat anti-rabbit IgG secondary antibody (Invitrogen, catalog no. 31460). For immunostaining of cells, we used the PhenoVu DNA Damage Response Staining Kit (revvity, catalog no. PDDR11), that contained primary antibodies (anti-Phospho H2AX (S139) – mouse IgG1, anti-53BP1 – rat IgG2a) and secondary antibodies (Fluor 488 – Goat anti-Mouse highly cross-adsorbed, Fluor 647 – Goat anti-Rat highly cross adsorbed).

Validation

Antibodies used were validated by the manufacturers for use in human cells.

Eukaryotic cell lines

Policy information about cell lines and Sex and Gender in Research

Cell line source(s)

409-B2 hiPSCs (Riken BioResource Center, HPS0076), Capan-1 cells (CLS cell line services, 300143), THP-1 (Cytion, catalog no. 300356), HEK293 cells (Cytion, catalog No. 300192), eHAP1 cells (Horizon, catalog No. C669), K562 cells (ECACC, catalog no. 89121407)

Authentication

All cell lines were authenticated by the supplier via certificate of analysis and additionally in-house by checking morphology.

Mycoplasma contamination

All cell lines were tested negative for mycoplasma contamination before and after the experiments.

Commonly misidentified lines (See ICLAC register)

No commonly misidentified cell lines were used in the study.

Plants

Seed stocks

not applicable

Novel plant genotypes

Describe the methods by which all novel plant genotypes were produced. This includes those generated by transgenic approaches, gene editing, chemical/radiation-based mutagenesis and hybridization. For transgenic lines, describe the transformation method, the number of independent lines analyzed and the generation upon which experiments were performed. For gene-edited lines, describe the editor used, the endogenous sequence targeted for editing, the targeting guide RNA sequence (if applicable) and how the editor was applied.

Authentication

Describe any authentication procedures for each seed stock used or novel genotype generated. Describe any experiments used to assess the effect of a mutation and, where applicable, how potential secondary effects (e.g. second site T-DNA insertions, mosiacism, off-target gene editing) were examined.
